# Supplementary material for: Thai Oakleaf Lettuce Phenocopies a Phytochrome B Mutant
Source: Biology (Basel). 2024 May 28;13(6):390. doi: 10.3390/biology13060390 (PMC11200548; doi:10.3390/biology13060390)
Supplement: Supplementary file 1 [file biology-13-00390-s001.zip › biology-2963962-supplementary.pdf]

Supplementary Data

Thai Oakleaf Lettuce Phenocopies a Phytochrome B Mutant

Cade Cooper and Kevin M. Folta

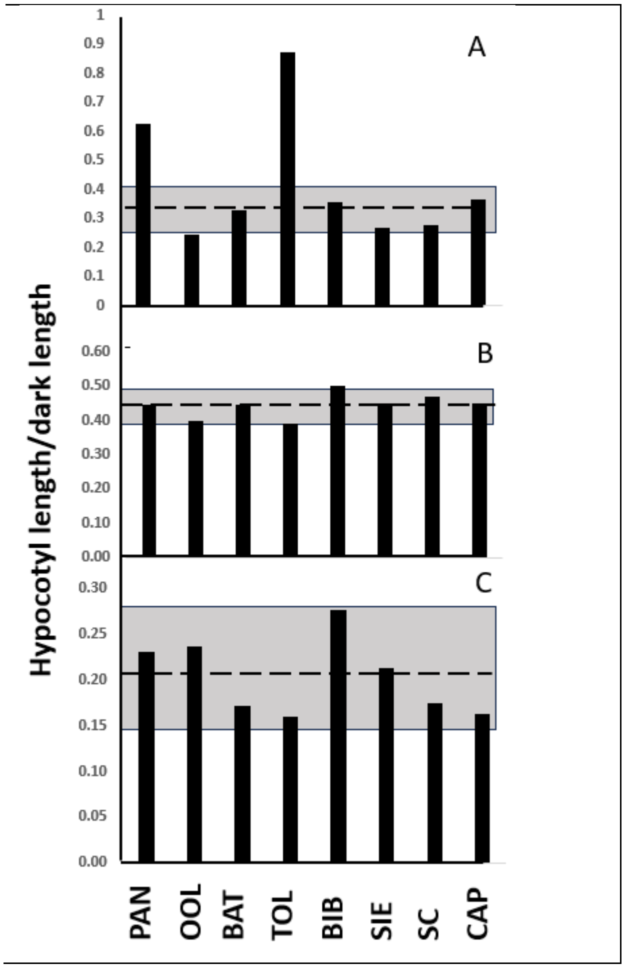

Supplemental Figure S1. Light response of reference genotypes to three spectral conditions. Seedlings from the specified genotypes were grown for 96 hours under red light (Panel A; 50 µmol/m^2s), blue light (Panel B; 5 µmol/m^2s) or cool white fluorescent light (Panel C; 20 µmol/m^2s) on vertical agar plates and measured. The results show the average length of at least 15 seedlings from at least two independent experiments, normalized to the genotype's dark growth rate. The dashed line represents the yet unpublished mean of 43 genotypes surveyed. The gray box indicates the 99% confidence interval for the entire set of 43 genotypes. Abbreviations: POL, Pannisse Oakleaf ; OOL, Oscarde Oakleaf; BAT, Batavian Crisphead, TOL, Thai Oakleaf;; BIB, Speckled ; SIE, Sierra Crisphead; SC, Summer Crisp Butterhead; CAP, Capitan.

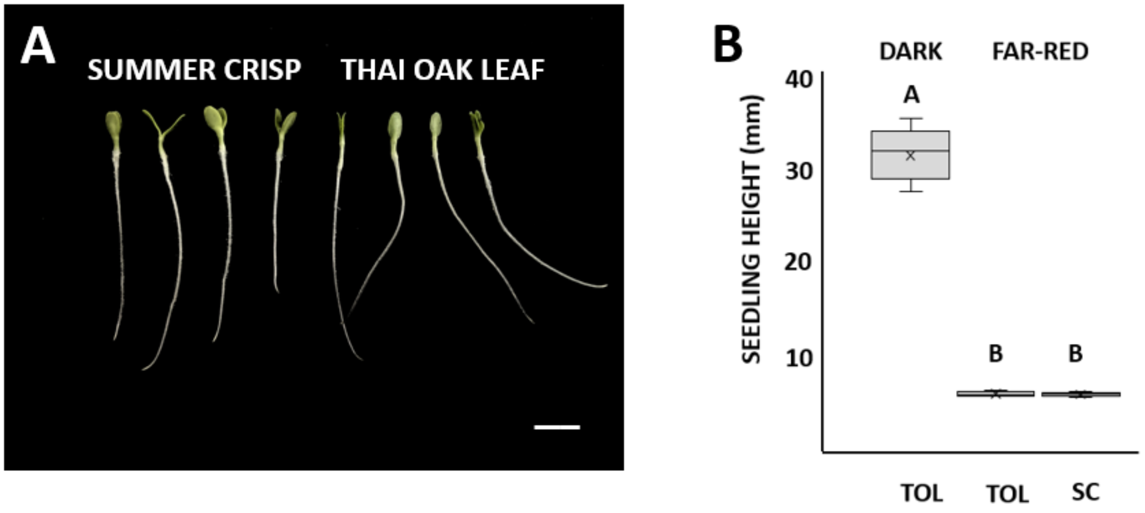

Supplemental Figure S2. Thai Oakleaf responds to far-red light. Seedlings from Thai Oakleaf (TOL) and Summer Crisp Butterhead (SC) were planted on media as described in Materials and Methods. Seeds were germinated in darkness for 48 h and then moved to far-red light at 35 µmol/m^{2}s for 96 h. The results show that both have similar responses to far-red light. Panel B presents the data from many seedlings compared to TOL in darkness. Letters represent significant differences at $p > 0.05$.
